# The Role of Glycolysis in Diabetic Kidney Disease

**DOI:** 10.3390/ijms27198793

**Published:** 2026-09-30

**Authors:** Yifan Wang, Xin Jiang, Liwen Cui, Yongke You, Wenjuan Zhu, Chongfei Jiang, Zhouning Liao, Jianbo Guo, Hao Lu, Baoyi Shao, Xiaozhou Luo, Pearl Pai, Haiyong Chen

**Affiliations:** 1 Division of Nephrology, Department of Medicine, The University of Hong Kong-Shenzhen Hospital, Shenzhen 518053, China; 2 School of Chinese Medicine, The University of Hong Kong, Hong Kong 999000, China; 3 Department of Nephrology, Shenzhen University General Hospital, Shenzhen University, Shenzhen 518055, China; 4 State Key Laboratory of Quantitative Synthetic Biology, Center for Synthetic Biochemistry, Shenzhen Institute of Synthetic Biology, Shenzhen Institutes of Advanced Technology, Chinese Academy of Sciences, Shenzhen 518055, China; 5 Department of Chinese Medicine, The University of Hong Kong-Shenzhen Hospital, Shenzhen 518053, China

**Keywords:** diabetic kidney disease, glycolysis, post-translational modification, HIF-1α, PKM2, SGLT2, epithelial–mesenchymal transition, podocyte, metabolic reprogramming

## Abstract

Diabetic kidney disease (DKD) is a leading cause of end-stage kidney disease worldwide and remains associated with high morbidity and mortality. In DKD, glycolytic reprogramming is triggered by hyperglycemia, hypoxia, oxidative stress, and inflammatory activation, which converge to drive metabolic imbalance. These stimuli promote excessive glycolytic flux, lactate accumulation, and diverse post-translational modifications, thereby reinforcing pathological reactions such as epithelial–mesenchymal transition, inflammation, and renal fibrosis. Accumulating evidence indicates that glycolytic alterations in DKD are highly cell type-specific. In podocytes, impaired glycolysis disrupts actin cytoskeletal integrity, promotes foot process fusion/effacement, and compromises the glomerular filtration barrier, ultimately leading to proteinuria. In tubular epithelial cells, glycolysis drives mesenchymal transformation and fibrogenesis, and engages in crosstalk with macrophages. In mesangial cells, glycolysis promotes mesangial cell hypertrophy and contributes to mesangial expansion. Mechanistically, central regulators including HIF-1α and PKM2, are modulated through glycolysis associated mechanisms, linking glycolysis to mitochondrial dysfunction, pathological cellular phenotypes, and DKD progression. Therapeutically, SGLT2 inhibitors have been clinically applied for DKD and kidney protection. Clinical studies further show that they can suppress maladaptive glycolytic activation in human metabolic analysis. Collectively, current evidence highlights glycolysis as a key driver of DKD and therapeutic targets for DKD management.

## 1. Introduction

### 1.1. Diabetic Kidney Disease

The prevention and management of diabetes mellitus (DM) and its complications remain serious challenges. The global prevalence of diabetes is as high as about 11%, affecting more than 580 million people. The prevalence of DM among people aged 20–79 is projected to reach approximately 13% by 2050 [1]. Global diabetes related healthcare spending has continued to rise significantly, leading to a heavy socioeconomic burden [2]. Diabetic kidney disease (DKD) is one of the most common complications of diabetes and is an important cause of chronic kidney disease (CKD). Approximately 20–40% of patients with type 1 DM (T1DM) or T2DM progress to DKD [3].

DKD is a progressive disease and one of the main causes of end-stage renal disease (ESRD). DKD is usually diagnosed clinically on the basis of albuminuria and/or reduced estimates glomerular filtration rate (eGFR) in individuals with diabetes, often without biopsy confirmation [4]. Renin–angiotensin–aldosterone system inhibitors (RAASi) have been the cornerstone therapy for DKD [5]. However, residual risk of kidney failure in patients treated with RAASi remains [6]. Several novel approaches are available to improve kidney outcomes in DKD patients: Sodium–glucose cotransporter 2 (SGLT2) inhibitors (Canagliflozin, dapagliflozin, and empagliflozin) [7,8,9], glucagon-like peptide-1 (GLP-1) receptor agonists (semaglutide) [10,11], selective endothelin receptor antagonist (atrasentan) [12], and non-steroidal mineralocorticoid receptor antagonists (finerenone) [13]. The pathomechanisms leading to DKD are very complex and include metabolic disorders, fibrosis, hemodynamic abnormalities, oxidative stress, inflammatory response, cell death, and genetic and epigenetic regulation [14,15,16]. Even though there have been many advances in DKD management, no existing drug therapies can completely reverse renal inflammation and fibrosis in DKD due to its complex pathology. Hence, it is vital to fully understand the pathomechanisms of DKD to allow discovery of new targets for early intervention and treatment.

### 1.2. Glycolysis in DKD

Metabolic reprogramming refers to the adaptive or maladaptive remodeling of cellular metabolic pathways in response to environmental stress, nutrient excess, hypoxia, mitochondrial dysfunction, inflammation, and altered energy demand [17]. In DKD, the kidney is exposed to persistent metabolic stress, including hyperglycemia, lipotoxicity, oxidative stress, local hypoxia, inflammatory activation, and profibrotic signaling. In response to these stresses, metabolic reprogramming involves coordinated changes in glucose metabolism, mitochondrial oxidative phosphorylation, fatty acid oxidation, amino acid metabolism, and lactate metabolism [18,19]. Among these alterations, the shift from mitochondrial oxidative metabolism to glycolysis is one of the most frequently observed metabolic changes, emerging as a central node integrating pathological signals in the progression of various diseases [20,21].

Glycolysis encompasses a ten-step cytosolic pathway that converts glucose to pyruvate, yielding two ATP molecules per glucose molecule. It is the first pathway for glucose breakdown and one of the major pathways for ATP generation present in all cell types. Glycolysis also produces various metabolic intermediates that functional in other pathways, such as pentose phosphate pathway, which supports biosynthesis. However, these intermediates can also accumulate under irregular glycolytic flux and contribute to pathological progress. Several key enzymes are tightly linked to the regulation of glycolysis, including hexokinase 2 (HK2) and pyruvate dehydrogenase kinase 1 (PDK1). Biological significance of glycolysis is shaped by cell type, disease mechanism, and experimental readout. In cancer, increased glycolytic flux is commonly framed as the Warburg effect, supporting proliferation, biosynthesis, and adaptation to the tumor microenvironment [22,23]. Cardiovascular and neuroinflammatory studies apply glycolysis as a marker of activated immune or stressed cellular states with an emphasis on lactate flux, glucose uptake, or pathway gene signatures [24,25,26]. In DKD, glycolysis is commonly interpreted as a major metabolic reprogramming process, regulated by varying expression levels of different glycolytic genes, and integrating various pathological signals that drive DKD progression. Hypoxia-inducible factor-1α (*HIF-1α*) is one of the most important genes that regulate glycolytic reprogramming. *HIF-1α* activates the glycolytic gene encoding *PDK*, thereby enhancing glucose conversion to pyruvate and lactate and facilitating metabolic adaptation under hypoxic conditions [27,28].

In DKD, altered glycolysis is not uniformly pathogenic. Its consequences depend on cell type, duration, metabolic demand, and mitochondrial function. A transient increase in glycolytic ATP production may compensate for impaired oxidative phosphorylation and preserve cellular viability or specialized functions [29]. However, glycolysis becomes maladaptive when persistent HIF-1α-dependent reprogramming is coupled to mitochondrial dysfunction, impaired pyruvate oxidation, and accumulation of lactate or reactive glycolytic intermediates [30]. These changes can promote oxidative stress, inflammation, protein lactylation, epithelial or endothelial transition, and fibrosis. Therefore, increased expression of glycolysis-related genes or proteins should not, by itself, be interpreted as evidence of pathogenic glycolytic flux.

Although the regulation of glycolytic gene expression and the corresponding enzymes is extensively characterized, the upstream triggers within the diabetic microenvironment that are associated with glycolytic reprogramming, both pathological and non-pathological, remain incompletely understood.

In this review, we synthesize the current understanding of glycolytic dysfunction in DKD, focusing on (1) the molecular mechanisms by which the diabetic microenvironment triggers glycolytic reprogramming, (2) the cell type-specific manifestations of glycolytic abnormalities, (3) glycolytic reprogramming as a source of potential biomarkers in DKD diagnosis, and (4) the therapeutic potential of targeting glycolytic enzymes. By integrating the diverse evidence of these different aspects, we aim to provide a comprehensive framework for understanding glycolysis as a central regulator of metabolic reprogramming and a promising target for DKD management.

## 2. Glycolytic Reprogramming in DKD

### 2.1. Hyperglycemia

Hyperglycemia, the defining feature of diabetes, imposes a direct glucotoxicity on renal cells. Glucose overload enhances glycolytic flux, leading to the accumulation of toxic intermediates such as dihydroxyacetone phosphate (DHAP), which triggers podocyte pyroptosis via mechanistic target of rapamycin complex 1 (mTORC1) signaling [31]. In response to hyperglycemia, UBR5 facilitates mesangial cell hypertrophy and glycolysis through increasing protein kinase B (AKT) phosphorylation [32]. Signaling through advanced glycation end-products (AGEs) and their receptor (RAGE) induced under hyperglycemia also amplifies glycolytic reprogramming [33]. AGE-RAGE ligation leads to generation of oxidative stress, which triggers inflammatory reactions through inducing proinflammatory cytokines [34]. Meanwhile, glycolysis generates AGE precursors, including methylglyoxal and glyoxal, further reinforcing the feedback loop of AGE-RAGE ligation and excessive glycolytic flux under hyperglycemia [35].

Hyperglycemia also diverts glucose into branch pathways. Polyol pathway flux increases fructose-derived DHAP, linking this branch pathway directly to glycolytic intermediates and PKC-β activation in DKD [36]. Increased GLUT1-mediated glucose flux in mesangial cells enhances de novo diacylglycerol synthesis, which activates PKC-β and drives downstream profibrotic signaling [37].

### 2.2. Hypoxia

Hypoxia represents another critical trigger in DKD. Renal tissue oxygen tension is reduced by glomerular hyperfiltration, capillary rarefaction, and increased oxygen consumption. Stabilization of HIF-1α initially constitutes an adaptive response to limited oxygen availability by inducing glycolytic enzymes and helping to preserve ATP production when mitochondrial oxidative phosphorylation is constrained [27]. However, the consequences of HIF-1α signaling depend on its magnitude and duration. Persistent activation can promote PDK-dependent restriction of mitochondrial pyruvate oxidation, sustained glycolytic reprogramming, and lactate accumulation, thereby reinforcing oxidative stress, inflammation, and fibrosis [27,28]. The effects of manipulating this pathway are correspondingly context dependent. In early-stage DKD, the HIF prolyl hydroxylase inhibitor Enarodustat reduces glycolytic metabolite accumulation [38], whereas SGLT2 inhibition suppresses a sustained HIF-1α-mediated shift from lipid oxidation to glycolysis in renal tubular cells [39]. COUP-TFII also attenuates HIF-1α-mediated glycolysis and promotes macrophage-to-myofibroblast transition [40]. Thus, transient HIF-dependent glycolysis may support adaptation to oxygen limitation, whereas persistent activation in the setting of mitochondrial dysfunction and inflammatory or profibrotic signaling may become maladaptive.

### 2.3. Oxidative Stress and Inflammation

Both hyperglycemia and hypoxia contribute to oxidative stress resulting in mitochondrial dysfunction. Under hyperglycemic conditions, cluster of differentiation 36 (CD36) activates the NOD-like receptor pyrin domain containing 3 (NLRP3) inflammasome via the mitochondrial reactive oxygen species (mtROS) pathway in renal tubular epithelial cells, driving mitochondrial damage that coincide with the metabolic shift from oxidative phosphorylation (OXPHOS) to glycolysis [41]. In renal fibrosis, upregulated G protein-coupled receptor 87 (GPR87) accelerates both glycolysis and mitochondrial injury, forming a feedback loop that enhances oxidative stress and exacerbates tubulointerstitial fibrosis [42]. Inflammatory cytokines also create a DKD-associated inflammatory microenvironment. These cytokines modulate glycolytic enzyme expression and activity, activate immune cells such as M1 macrophages, and promote glycolysis through *HIF-1α–HK2* signaling [43].

**Figure 1:**
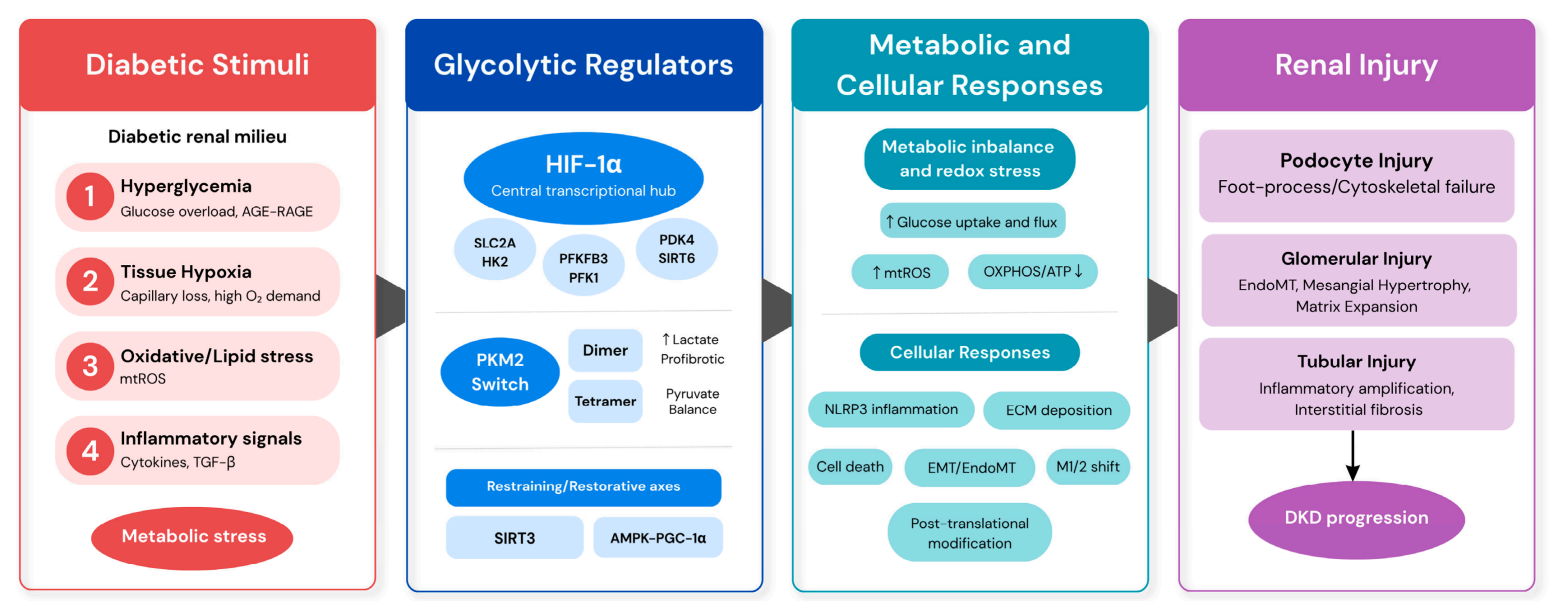
Glycolytic dysregulation and renal injury in diabetic kidney disease. Abbreviations: AGE, advanced glycation end-product; AMPK, AMP-activated protein kinase; ECM, extracellular matrix; EMT, epithelial–mesenchymal transition; EndoMT, endothelial–mesenchymal transition; HIF-1α, hypoxia-inducible factor-1α; HK2, hexokinase 2; mtROS, mitochondrial reactive oxygen species; M1/2 shift, macrophage M1/M2 shift; NLRP3, NOD-like receptor protein 3; OXPHOS, oxidative phosphorylation; PDK4, pyruvate dehydrogenase kinase 4; PFK1, phosphofructokinase 1; PFKFB3, 6-phosphofructo-2-kinase/fructose-2,6-bisphosphatase 3; PGC-1α, peroxisome proliferator-activated receptor-γ coactivator-1α; PKM2, pyruvate kinase M2; SIRT3, sirtuin-3; SIRT6, sirtuin-6; SLC2A, solute carrier family 2.

Diabetic stimuli activate key glycolytic regulators, driving metabolic imbalance and multiple post-translational modifications (PTMs) that contribute to progressive glomerular and tubular injury, as shown in Figure 1.

## 3. Pathological Mechanisms of Glycolysis in DKD

**Figure 2:**
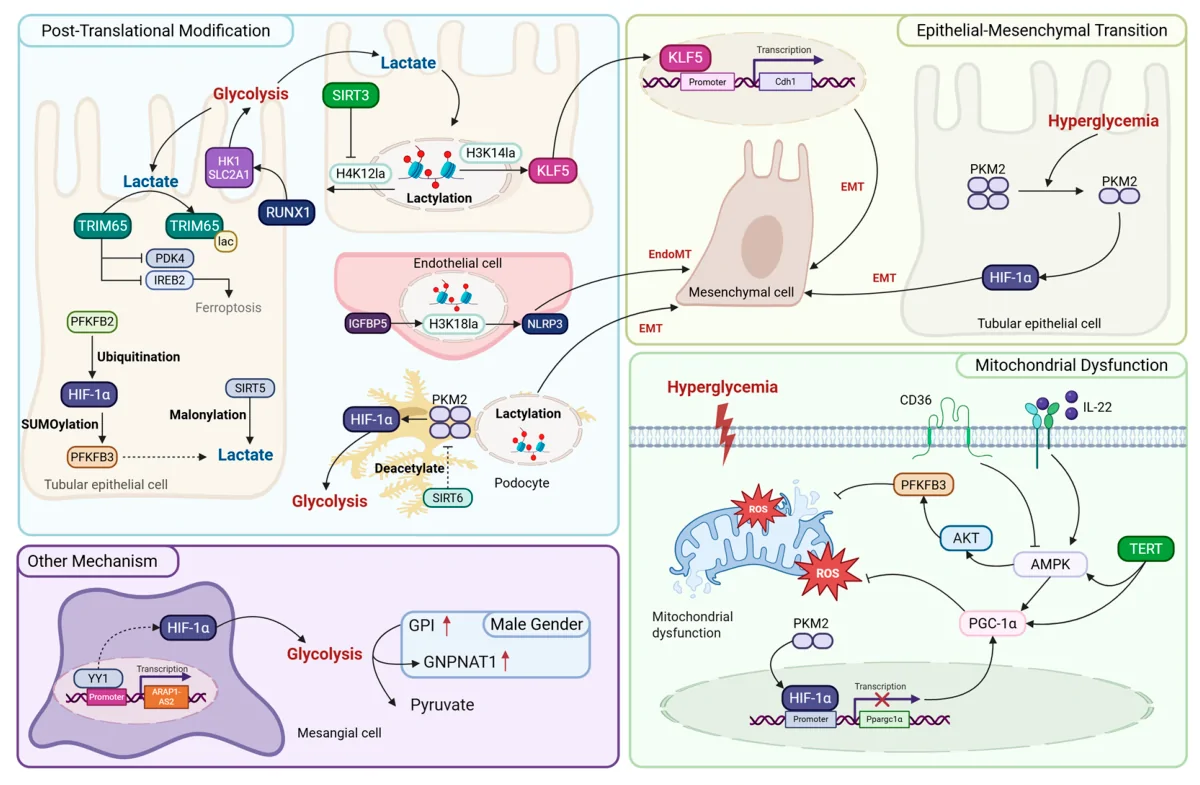
Summarized mechanisms of glycolysis in DKD. Abbreviations: AKT, protein kinase B; AMPK, AMP-activated protein kinase; *ARAP1-AS2*, Arfgap with rhogap domain, ankyrin repeat and ph domain 1-antisense RNA 2; CD36, cluster of differentiation 36; DENV-2, dengue virus serotype 2; EMT, epithelial–mesenchymal transition; EndoMT, endothelial-to-mesenchymal transition; GPI, glucose-6-phosphate isomerase; GNPNAT1, glucosamine-phosphate N-acetyltransferase 1; H3K14la, histone H3 lysine 14 lactylation; H3K18la, histone H3 lysine 18 lactylation; H4K12la, histone H4 lysine 12 lactylation; HIF-1α, hypoxia-inducible factor 1-alpha; HK1, hexokinase 1; IREB2, iron-responsive element-binding protein 2; IGFBP5, insulin-like growth factor binding protein 5; IL-22, interleukin-22; KLF5, Krüppel-like factor 5; Malonylation, lysine malonylation modification; NLRP3, NOD-, LRR- and pyrin domain-containing protein 3; PDK4, pyruvate dehydrogenase kinase 4; PFK1, phosphofructokinase 1; PFKFB2, 6-phosphofructo-2-kinase/fructose-2,6-bisphosphatase 2; PFKFB3, 6-phosphofructo-2-kinase/fructose-2,6-bisphosphatase 3; PKM2, pyruvate kinase M2; PGC-1α, peroxisome proliferator-activated receptor gamma coactivator 1-alpha; *Ppargc1a*, peroxisome proliferator-activated receptor gamma coactivator 1-alpha gene; ROS, reactive oxygen species; RUNX1, runt-related transcription factor 1; SIRT3, sirtuin 3; SIRT5, sirtuin 5; SIRT6, sirtuin 6; SLC2A1, solute carrier family 2 member 1; SUMOylation, small ubiquitin-like modifier conjugation; TERT, telomerase reverse transcriptase; TRIM65, tripartite motif-containing protein 65; YY1, Yin Yang 1 transcription factor.

Multiple glycolytic mechanisms regulate the pathology in DKD, as summarized in Figure 2.

### 3.1. Post-Translational Modification

Glycolytic remodeling in DKD is initiated by multiple upstream regulators, including lactylation, ubiquitination, SUMOylation, malonylation and deacetylation. These mechanisms act as primary drivers that shift glucose metabolism toward glycolysis and set the stage for downstream cellular responses. Metabolites from glycolysis promote various types of protein PTMs, including acetylation, methylation, ubiquitination, hydroxylation, SUMOylation, phosphorylation, glycosylation, and others [44,45].

#### 3.1.1. Lactylation

During the complex process of glucose oxidative metabolism, pyruvate serves as a key intermediate. Under the catalytic activity of lactate dehydrogenase (LDH), pyruvate is further converted into lactate [46]. As the major product of glycolysis, lactate promotes histone lactylation in DKD. Under DKD conditions, lactylation drives EMT by increasing histone H3 lysine 14 lactylation (H3K14la) and subsequently activating Krüppel-like factor 5 (KLF5) transcription in renal tubular epithelial cells. In db/db mouse model, blocking the lactate/H3K14la/KLF5 signaling cascade has been shown to alleviate renal injury and DKD progression [47]. Elevated lactate levels also trigger histone H4K12 lactylation (H4K12la), an epigenetic alteration that significantly enhances RUNX family transcription factor 1 (RUNX1) transcription. Upregulated RUNX1 further induces the expression of the glycolytic genes *HK1* and solute carrier family 2 member 1 (*SLC2A1*). SIRT3 has been identified as a delactylase and a regulator of cellular lactate metabolism, and its deficiency contributes to metabolic dysregulation in DKD [48]. SIRT3 downregulation triggers activation of transforming growth factor beta (TGF-β)/Smad signaling, which is accompanied by increased glycolysis-related HIF-1α accumulation and pyruvate kinase isoform M2 (PKM2) dimerization [49]. Overexpression of SIRT3 deacetylates RUNX1, reducing its transcriptional activity, and consequently limits H4K12la [48].

Other PTMs also regulate protein expression levels and activity. Insulin-like growth factor-binding protein 5 (IGFBP5) is a key secreted protein closely associated with tissue inflammation and fibrosis. IGFBP5 is upregulated in DKD and promotes EndoMT and renal fibrosis through H3K18 lactylation [50]. IGFBP5 knockdown effectively blocks high-glucose-triggered endothelial-to-mesenchymal transition (EndoMT) specifically induced by glycolysis-mediated NLRP3 inflammasome activation in glomerular endothelial cells. Mechanistically, H3K18la acts as an epigenetic modification driven by high cellular glycolysis and lactate accumulation that increases chromatin accessibility at the promoters of inflammatory genes, thereby enhancing their transcription and promoting the production of inflammatory proteins including NLRP3. IGFBP5 knockdown decreases histone lactylation and the subsequent transcription, inhibiting NLRP3 inflammasome production and EndoMT [50].

#### 3.1.2. Ubiquitination

Tripartite motif-containing 65 (TRIM65) plays a renoprotective role in DKD by regulating ubiquitination, ferroptosis, and glycolysis [51]. TRIM65 suppresses renal ferroptosis by degrading iron-responsive element-binding protein 2 (IREB2) and mitigates diabetic renal injury by downregulating pyruvate dehydrogenase kinase 4 (PDK4) to inhibit excessive glycolysis [51]. Lactate induces lactylation of TRIM65 at K206, which inactivates TRIM65 and abolishes its protective functions [51]. 6-phosphofructo-2-kinase/fructose-2,6-bisphosphatase 2 (PFKFB2) emerges as a critical regulator of glycolysis and oxidative stress [52]. PFKFB2 expression is significantly downregulated in tissues from patients with DKD. Loss of PFKFB2 aggravates disease progression by enhancing glycolysis, oxidative stress, and mitochondria-dependent ferroptosis. These studies highlight the interplay between ubiquitination, glycolysis, and ferroptosis.

#### 3.1.3. SUMOylation

SUMOylation is a reversible PTM mediated by small ubiquitin-like modifier (SUMO) proteins, which regulates protein stability, activity, localization, and interaction networks [53]. SUMO-activating enzyme subunit 1 (SAE1) is upregulated in diabetic kidneys. Reduced SAE1 expression decreases HIF-1α and PFKFB3 levels, thereby correcting abnormal glycolysis and mesenchymal transformation. Mechanistically, SAE1-dependent SUMOylation stabilizes HIF-1α, which transcriptionally activates PFKFB3 and enhances renal glycolysis, as reflected by increased PFK1 activity and lactate accumulation. Inhibition of PFKFB3 alleviates renal fibrosis, whereas its overexpression partially rescues glycolysis and mesenchymal transformation suppressed by SAE1 knockdown [54].

#### 3.1.4. Malonylation

Malonylation represents a conserved acylation event across evolution, and its dysregulation has been implicated in diverse metabolic disorders, including inflammatory conditions, type 2 diabetes, and aberrant angiogenesis [55]. Decreased renal cortical malonylation occurs alongside SIRT5 upregulation in db/db mice kidney. Elevated SIRT5 expression is consistently detected in the tubulointerstitium of human DKD kidneys. Decreased SIRT5 suppresses glycolytic activity, linking SIRT5-mediated malonylation deficiency to enhanced glycolysis in DKD [56].

#### 3.1.5. SIRT6-Mediated Deacetylation and Glycolytic Restraint

SIRT6 also plays a pivotal role in restraining glycolysis by inhibiting the transcriptional activity of HIF-1α. SIRT6 functions as a histone H3K9 deacetylase to suppress glycolytic gene transcription and maintain a balance in glucose utilization through the tricarboxylic acid cycle [57]. In SIRT6-deficient cells, HIF-1α protein stability is enhanced, leading to overexpression of a range of enzymes encoded by glycolytic genes, including lactate dehydrogenase, triose phosphate isomerase, aldolase, and phosphofructokinase**,** which drive excessive glycolytic flux. Sirt6 knockout mouse shows enhanced glycolytic gene expression, including PDK1 and phosphofructokinase-1 (PFK-1), and drive excessive lactate production [58]. However, this study was performed in embryonic stem cells and mouse embryonic fibroblasts rather than renal cells, and did not demonstrate kidney-specific effects of SIRT6 on glucose metabolism, so its relevance to DKD remains indirect.

Therefore, these findings establish a broadly applicable SIRT6–HIF-1α regulatory mechanism but do not demonstrate that SIRT6 directly restrains glycolysis in renal cells (podocytes, tubular epithelial cells, mesangial cells, or glomerular endothelial cells) during DKD. Although HIF-1α is known to regulate glycolysis, whether SIRT6 modulates glycolytic activity in renal cells remains undiscovered.

In addition to deacetylation of H3K9, SIRT6 can also deacetylate PKM2. In DKD, podocyte-specific overexpression of PKM2 protects mitochondrial function by activating the HIF-1α/vascular endothelial growth factor (VEGF) pathway, mitigating hyperglycemic toxicity and slowing disease progression [59].

### 3.2. Interactions Between Glycolytic Reprogramming and Mitochondrial Dysfunction

Mitochondrial dysfunction and glycolytic reprogramming reinforce each other in DKD, forming an interconnected metabolic axis rather than independent parallel processes. Mitochondrial dysfunction occurs in both non-diabetic and diabetic renal tissues and is characterized by impaired respiratory-chain activity, disrupted mitochondrial structure and network organization, dysregulated cellular signaling, and excessive reactive oxygen species production [60]. The accompanying glycolytic response is stage dependent. A transient increase in glycolytic ATP production may compensate for an acute decline in mitochondrial respiration and help maintain cellular energy balance. If mitochondrial function fails to recover, however, persistent glycolytic reprogramming may become maladaptive through impaired pyruvate oxidation, redox imbalance, and lactate accumulation. In DKD, mitochondrial defects are associated with elevated HIF-1α and reduced peroxisome proliferator-activated receptor-γ coactivator-1α (PGC-1α) expression, renal fibrosis, and cellular apoptosis [60,61]. Mechanistically, HIF-1α directly targets the *Ppargc1a* promoter, and PKM2 negatively regulates this interaction. Sustained HIF-1α signaling represses PGC-1α expression and promotes abnormal energy metabolism, mitochondrial dysfunction, and apoptosis, whereas inhibition of this pathway reverses these changes [62]. These findings also emphasize that the effect of PKM2 depends on its functional state: catalytically active PKM2 can support pyruvate production and mitochondrial homeostasis, whereas persistent HIF-1α-linked glycolytic signaling may be injurious. Telomerase reverse transcriptase (TERT) deficiency further disrupts renal mitochondrial homeostasis under hyperglycemic and hyperlipidemic stress [63]. Reduced TERT expression causes AMP-activated protein kinase (AMPK) dephosphorylation, impaired mitochondrial complex activity and ATP production, and disruption of the nicotinamide adenine dinucleotide (NAD)+/NADH balance, leading to excess ROS generation and lipid accumulation in renal tubular cells [64]. Conversely, TERT overexpression ameliorates renal mitochondrial dysfunction through the AMPK/PGC-1α axis [64].

Interleukin-22 (IL-22) provides an example in which enhancement of a glycolysis-related pathway is associated with protection rather than injury. In damaged renal tubular epithelial cells (TECs), IL-22 activates AMPK/AKT signaling and enhances PFKFB3 activity while preserving mitochondrial integrity and reducing mitochondrial ROS accumulation [65,66]. This finding suggests that increased glycolytic capacity can be compensatory when it is coordinated with maintenance of mitochondrial function. In contrast, upregulated CD36 promotes mitochondrial ROS overproduction, NLRP3 inflammasome activation, caspase-1 cleavage, IL-1β secretion, and tubular-cell apoptosis in DKD [67]. CD36 deficiency enhances AMPK activity and restores fatty-acid oxidation, thereby suppressing mitochondrial ROS generation and NLRP3-mediated inflammation [41].

Hyperglycemia induces mitochondrial damage and bioenergetic deficits in a cell-type-specific manner [68]. Compared with human primary mesangial cells (HMCs), human proximal tubular cells (HK-2) exhibit lower basal, ATP-coupled, and maximal respiration, as well as lower respiratory reserve, under normoglycemic conditions [68]. Short-term hyperglycemia selectively impairs tubular mitochondrial respiration, whereas mesangial cells remain metabolically stable. After four days of hyperglycemic exposure, tubular cells develop mitochondrial dysfunction and activate compensatory glycolysis to offset the energy deficit [68]. This response may initially preserve ATP availability, but its persistence without mitochondrial recovery could become maladaptive. In contrast, 12 days of hyperglycemic exposure gradually compromises mitochondrial bioenergetics in HMCs without inducing glycolytic upregulation [68]. Together, these observations show that the biological meaning of altered glycolysis depends on cell type, duration of stress, and whether the response restores energy balance or remains coupled to persistent mitochondrial failure. Longitudinal studies are needed to define when compensatory glycolysis transitions to a maladaptive state in DKD.

### 3.3. Downstream Phenotypic Consequences: EMT and Tubular Maladaptation

The metabolic and mitochondrial alterations described above converge on downstream phenotypic changes, most prominently partial EMT and tubular maladaptation. Epithelial–mesenchymal transition (EMT) is a fundamental biological process in which epithelial cells undergo phenotypic transformation into mesenchymal cells [69]. EMT is essential for embryonic development and tissue repair, and EMT-like phenotypic changes are frequently observed in chronic kidney injury [70]. In DKD, tubular epithelial cells may acquire mesenchymal features and gradually lose aspects of their reabsorptive and secretory functions. Whether these changes represent a full EMT or only a partial shift in cell state is still under investigation. Current evidence indicates that such partial EMT contribute to tubular dysfunction, matrix accumulation and tissue remodeling, which together promote the progression of renal fibrosis [71].

As mentioned above, H3K14la is a critical epigenetic regulator of glycolysis in DKD. It has been known that H3K14la promotes EMT by upregulating KLF5 expression in db/db mice and tubular epithelial cells. Mechanistically, KLF5 binds to the promoter region of Cdh1 and suppresses its transcription, thereby driving tubular EMT and worsening DKD progression. In DKD models, nephron-specific KLF5 knockdown and pharmacological KLF5 inhibition block EMT activation and alleviate renal fibrosis effectively [47]. In renal tubular HK-2 cells, pharmacological PKM2 activation by TEPP-46 efficiently inhibits EMT activation induced by TGF-β1 stimulation and hyperglycemic stress. TEPP-46 facilitates PKM2 tetramer formation and restores pyruvate kinase activity. This process inhibits HIF-1α accumulation and reduces lactate production [72]. Apart from EMT of renal tubular under diabetic conditions, podocytes also undergo prominent EMT in DKD progression. Reducing lactate accumulation efficiently represses histone lactylation, reverses podocyte EMT, and alleviates DKD-related renal damage [73].

### 3.4. Additional Mechanisms Associated with Glycolytic Dysregulation

Several additional mechanisms complement the core glycolytic axis in DKD and contribute to metabolic imbalance and renal injury. Yin and Yang 1 (YY1) is known to serve as a therapeutic target for early tubulointerstitial fibrosis in DKD [74]. YY1 upregulates long non-coding RNA (lncRNA) ArfGAP with RhoGAP domain, ankyrin repeat and PH domain 1 (ARAP1)-antisense RNA 2 (AS2) and subsequent ARAP1 expression, triggering persistent epidermal growth factor receptor overactivation in renal tissues [75]. This molecular cascade remodels PKM2 conformation by promoting dimeric PKM2 accumulation and impairing tetrameric PKM2 formation, which induces HIF-1α aggregation, aberrant glycolytic reprogramming, and profibrotic gene activation. Consistently, ARAP1 inhibition consistently reverses glycolytic dysfunction, suppresses renal fibrosis, and ameliorates renal injury in DKD, and validates the role of the YY1/*ARAP1-AS2*/ARAP1 axis in driving early DKD progression [75].

Sex differences appear to influence the progression of DKD, with male sex representing a risk factor for accelerated renal injury [76]. Androgen signaling, particularly through dihydrotestosterone (DHT), regulates renal energy metabolism and contributes to oxidative stress in tubular cells in acute kidney injury (AKI) [77]. Quantitative proteomic analyses of human proximal tubular epithelial cells reveal that DHT selectively regulates proteins involved in glycolysis and related metabolic pathways [78]. Notably, glucose-6-phosphate isomerase (GPI) is consistently upregulated in male kidneys. Compared with females, male diabetic mice exhibit significantly higher expression of GPI, glucosamine-phosphate N-acetyltransferase 1 (GNPNAT1), and fatty acid β-oxidation related hydroxyacyl-CoA dehydrogenase (HADHA), linking androgen-driven metabolic reprogramming to DKD pathogenesis. Enrichment analyses further associate DHT-regulated proteins with enhanced oxidative stress, a hallmark of DKD progression. Collectively, these findings suggest that male sex hormones aggravate DKD by promoting glycolytic activation and metabolic imbalance, thereby intensifying oxidative stress and structural damage in the kidney.

## 4. Cell-Specific Glycolysis in DKD

Cell type-specific responses to the diabetic microenvironment add further complexity to the glycolytic landscape. Podocytes rely on both glycolysis and oxidative phosphorylation for basal energy production, but glycolysis predominates in supporting cortical ATP distribution and cytoskeletal dynamics essential for filtration [79]. ATP generated through glycolysis primarily supports cytoskeletal dynamics in cortical regions of podocytes, and sustain structural and functional activity [80]. GScore is developed through Single cell RNA-seq of diabetic kidney, which is an integrative machine learning derived diagnostic model constructed from glycolysis related genes, enabling early prediction and risk stratification of DKD. It reflects glycolysis associated transcriptional alterations and immune microenvironment changes, providing a potential biomarker framework for DKD diagnosis [81].

### 4.1. Podocytes

Podocytes rely predominantly on glycolysis for energy production and exhibit a unique vulnerability. Any compromise in glycolysis contributes to foot process fusion [82,83]. PKM2 is a key regulator of glycolysis in podocytes. Podocytes exhibit reduced expression of PKM2 in biopsy samples from patients with DKD [82]. However, in the glomeruli of DKD mouse models or in mouse podocytes treated with high glucose, no decrease in PKM2 protein expression has been observed [84]. Mechanistically, hyperglycemia impairs podocyte metabolism by shifting PKM2 from active tetramers to less active dimers and monomers, reducing the enzymatic activity, thereby weakening glycolytic capacity of glomeruli [85]. The podocyte-specific PKM2 knockout in diabetic mice enhances albuminuria and glomerular injury, and is accompanied by a compensatory increase in PKM1 expression. However, compensatory upregulation of PKM1 or pyruvate kinase (PK) activity fails to protect against DKD, possibly as a result of distinct mitochondrial regulatory roles of PKM1 and PKM2 [84]. Notably, hyperglycemia does not alter PK activity in mesangial or endothelial cells [84].

Another key regulator of podocyte metabolic and immune homeostasis is the signal regulatory protein alpha (SIRPα). Loss of SIRPα aggravates podocyte injury in DKD, whereas its overexpression alleviates renal damage. Deficiency of SIRPα enhances PKM2 phosphorylation, which in turn drives its translocation into the nucleus in podocytes, thereby enhancing the NF-κB signaling and inflammation [86]. Hyperglycemia drives the translocation of Smad4 to the mitochondria, where it interacts with PKM2 and ATP synthase inhibitory factor subunit 1 (ATPIF1) to suppress glycolysis and oxidative phosphorylation while increasing ROS, thereby inhibiting formation of the active PKM2 tetramer. In contrast, Smad4 depletion enhances energy metabolism and protects against glomerulosclerosis [87]. Canonical TGF-β1/Smad4 signaling typically involves Smad4 translocation to the nucleus, where it regulates gene transcription [88]. However, regulation of glycolysis by Smad4 in podocytes occurs independent of its nuclear function [87]. In podocytes, ornithine catabolism is further exaggerated in the absence of active PKM2. Pharmacological inhibition of ornithine decarboxylase with difluoromethylornithine reduces albuminuria and improves podocyte morphology in diabetic mice. It follows that putrescine derived from ornithine catabolism activates Rheb and mTOR signaling, driving cytoskeletal remodeling [79].

Glycolytic metabolite accumulation contributes to podocyte-related glycolysis. Dysregulated glycolytic enzyme expression in DKD leads to altered fructose-1,6-bisphosphate (FBP) metabolism, and drives podocyte cytoskeletal remodeling and renal injury. Restoration of metabolic balance through modulation of phosphofructokinase platelet type (PFKP)/aldolase type B (ALDOB) or exogenous FBP supplementation protects against podocyte damage and glomerular pathology [89]. In db/db mouse model, elevated DHAP emerges as a critical metabolic driver of podocyte pyroptosis in DKD, alongside with the decline of lactate dehydrogenase A (LDHA) and PKM2. ALDOB upregulation amplifies DHAP levels, leading to mTORC1 activation, oxidative stress, and cytoskeletal damage. Importantly, rapamycin suppresses mTORC1-mediated ROS and pyroptosis [31]. Based on these studies, ALDOB may act as a potential therapeutic target for podocyte metabolite accumulation in DKD.

### 4.2. Mesangial Cells

The E3 ubiquitin ligase UBR5 emerges as an important regulator of mesangial cell metabolism and hypertrophy in DKD. UBR5 expression is consistently elevated in diabetic mouse models and HMCs under high-glucose conditions [32]. Functionally, UBR5 facilitates HMC hypertrophy and glycolysis through activation of the AKT pathway, establishing a direct link between metabolic reprogramming and structural damage. Silencing UBR5 mitigates these pathological changes, while upstream regulation via WTAP-mediated m6A modification and IGF2BP1 stabilization further enhances UBR5 expression [32].

### 4.3. Endothelial Cells

Diabetes-induced glomerular endothelial cell (GEC) dysfunction is characterized by the emergence of proinflammatory and prothrombotic phenotypes, which promote immune cell adhesion and infiltration, disrupt fenestrated endothelial integrity, and accelerate endothelial-to-mesenchymal transition [90]. IGFBP5 and PFKFB3 are key regulators of glycolysis in endothelial cells. IGFBP5 is markedly elevated in DKD and plays a pathogenic role in driving endothelial cell inflammation. Loss of IGFBP5 alleviates renal inflammatory injury, while its presence enhances glycolysis through activation of early growth response 1 and induction of PFKFB3 in human umbilical vein endothelial cells [91]. IGFBP5 serves as a positive regulator of aerobic glycolysis, resulting in a pronounced increase in both lactate levels and the extracellular acidification rate. PFKFB3 mutation can counteract IGFBP5 and attenuates renal inflammation [91]. As previously described, IGFBP5 is involved in EndoMT. Knockdown of IGFBP5 restores endothelial identity by upregulating CD31 and VE-cadherin, while simultaneously reducing mesenchymal markers such as alpha-smooth muscle actin (α-SMA) and fibroblast-specific protein-1 (FSP-1) found in DKD. The same IGFBP5 study also reported reductions in LDHA and PKM2 expression following IGFBP5 inhibition, indicating that IGFBP5 intersects with glycolytic regulation [50].

Hyperglycemia enhances glycolysis and upregulates PFKFB3 in GEC, which drive a proangiogenic phenotype. Overexpression of miR-590-3p suppresses PFKFB3, while simultaneously increasing sphingosine 1-phosphate receptor 1 (S1PR1) expression, thereby attenuating endothelial migration and angiogenic potential. The pharmacological or genetic inhibition of PFKFB3 also upregulates S1PR1 and reinforces the regulatory link between glycolysis and angiogenesis. Diabetic mice with reduced miR-590-3p in the renal cortex develop albuminuria and disrupted endothelial fenestration. Restoration of miR-590-3p expression rescues endothelial fenestrations and improves renal function [92].

### 4.4. Tubular Epithelial Cells

Renal tubules and the surrounding tubulointerstitium constitute the largest compartment of the kidney and represent the primary sites of injury response [93]. As described above, in the presence of DKD, glycolysis in TECs is regulated by multiple factors. An interesting crosstalk between TECs and macrophages has been observed. Extracellular vesicles (EVs) are released from human serum albumin (HSA)-stimulated TECs. This promotes macrophage glycolysis through stabilizing HIF-1α and subsequently enhances inflammatory and fibrotic gene expression. Mechanistically, EVs derived from HSA-stimulated TECs accelerate glycolysis through HIF-1α stabilization, thereby linking epithelial injury to macrophage metabolic reprogramming and inflammation in DKD [94].

Senescent cells, characterized by elevated expression of the cyclin-dependent kinase inhibitor p16, are increasingly recognized as important regulators [95]. Significantly upregulated senescence markers are observed in human with DKD, streptozotocin (STZ)-induced diabetic mice, and HK-2 cells cultured under high glucose conditions. Selective clearance of p16-positive cells in INK-ATTAC mice reduces senescence-associated secretory phenotype, restores ATP content, and suppresses glycolytic gene expression via AMPK–mTOR signaling. Mechanistically, p16 mediates cell cycle arrest through the CDK4–Rb pathway, linking tubular epithelial cell senescence to metabolic dysfunction [96].

### 4.5. Macrophages

Macrophages are central players in immune regulation, with distinct functional phenotypes that determine inflammatory outcomes. Classically activated M1 macrophages, driven by Toll-like receptor and interferon signaling, are primarily associated with proinflammatory responses and defense against bacteria and intracellular pathogens [97]. Alternatively activated M2 macrophages, being induced in a T helper 2 (Th2)-dominated environments such as helminth infection, asthma, and allergy, contribute to anti-inflammatory processes and tissue repair [98]. However, the definition of M1 and M2 remains controversial as macrophage phenotypes lack uniformity in their classification.

In DKD, macrophages exhibit a shift toward the proinflammatory M1 phenotype, accompanied by enhanced glycolytic activity, while HIF-1α plays a vital role in regulating glycolysis in M1 macrophages [43]. M1 macrophage polarization enhances glycolysis, alongside elevated levels of HIF-1α and HK2. Mechanistically, HIF-1α drives M1 polarization and glycolysis by activating HK2 transcription [43]. Beyond the HIF-1α/HK2 axis that drives glycolytic reprogramming in M1 macrophages, epigenetic regulation via m6A/Npas2/Hif-1α also plays a pivotal role. The m6A demethylase Fto is markedly reduced in renal macrophages from db/db mice. Fto controls the stability of Npas2 transcripts through a Prrc2a-dependent mechanism, thereby regulating M1 polarization and glucose metabolism via Hif-1α signaling [99]. As a nuclear receptor, COUP-TFII participates in metabolism and immune evasion [100]. In DKD, TGF-β signaling upregulates COUP-TFII, which in turn increases profibrotic markers such as α-SMA and collagen I, particularly in macrophages. Knockdown of COUP-TFII reduces these fibrotic proteins but enhances HIF-1α, HK2, and PKM2 expression, suggesting a link between fibrosis and glycolytic reprogramming [40].

While M1 macrophages drive proinflammatory responses and glycolytic activation in DKD, M2 macrophages behave as the counterbalancing phenotype, associated with anti-inflammatory activity and tissue protection. Recent studies highlight that metabolic regulation, particularly through Sirt6, plays a decisive role in promoting M2 polarization and mitigating podocyte injury under high-glucose conditions [101]. Sirt6 overexpression shifts macrophages toward the M2 phenotype, characterized by increased CD206 and Bcl-2 expression and reduced CD86 and Bax levels [101]. In co-culture systems, while Sirt6-induced M2 macrophages protect podocytes from high-glucose-induced apoptosis, podocyte-specific Sirt6 overexpression alone does not confer protection. Mechanistically, Sirt6 attenuates glycolysis and inflammatory signaling in macrophages, thereby reprogramming their phenotype toward anti-inflammatory M2 cells [101]. These findings suggest that Sirt6 functions as a metabolic and immune regulator, linking M2 macrophage glycolysis suppression to renal protection in DKD. Another study highlights how mesenchymal stem cells regulate macrophage metabolism and phenotype [102]. In co-culture systems, MSC-derived mitochondria are delivered into macrophages, enhancing mitochondrial function and driving M2 polarization. This process activates PGC-1α-mediated mitochondrial biogenesis and interacts with TFEB to promote lysosomal autophagy, facilitating the clearance of damaged mitochondria. As a result, macrophages exhibit improved bioenergetics and anti-inflammatory capacity, while knockdown of PGC-1α abolishes these protective effects [102]. Although this study does not directly measure glycolysis-related indicators, the central mediator, PGC-1α, is a downstream target of HIF-1α tightly linked to glycolytic regulation. This suggests that MSC-mediated M2 polarization intersect with glycolysis in DKD.

## 5. Therapeutic Strategies Targeting Glycolysis, Glycolysis-Related Pathways, and Metabolites in DKD

Multiple strategies have been applied for the treatment of glycolysis in DKD, summarized in Table 1.

### 5.1. SGLT2 Inhibitors

Accumulating clinical evidence indicates that SGLT2 inhibitors exert unexpected renoprotective actions in DKD [103]. Clinical intervention with canagliflozin, a SGLT2 inhibitor, lead to metabolic benefits in DKD patients. A 12-week treatment significantly reduces glycolysis and enhances fatty acid oxidation. In a clinical cohort of patients with type 2 DKD who received 100 mg per day of canagliflozin for twelve weeks, metabolomic profiling of plasma samples from 35 participants revealed a coordinated metabolic shift. Glycolytic flux was suppressed and fatty acid oxidation was enhanced, and targeted analyses further showed a significant increase in circulating glycine levels [104]. Canagliflozin also upregulates glycine, serine and threonine metabolism in db/db mice. Glycine supplementation further ameliorates renal lesions by improving insulin sensitivity, reducing blood glucose and attenuating podocyte apoptosis through the AMPK–mTOR pathway [104].

Dapagliflozin is a potent and reversible SGLT2 inhibitor that is widely approved for the treatment of type 2 diabetes [105]. Dapagliflozin alleviates diabetes-induced tubulointerstitial damage, including macrophage infiltration and fibrosis. Importantly, dapagliflozin nearly abolishes elevated HIF-1α levels in renal proximal tubules. Mechanistically, dapagliflozin protects against glucose-induced metabolic shift in proximal tubular cells by inhibiting HIF-1α, thereby preventing aberrant glycolysis and restoring metabolic homeostasis [39]. Another study evaluates dapagliflozin in an AKI–DKD model, where metabolic reprogramming is characterized by impaired fatty acid oxidation and enhanced glycolysis. Dapagliflozin treatment improves abnormal glycolysis via restoration of SIRT3 expression [106]. This study uses a diabetic ischemia–reperfusion injury model to mimic the AKI–CKD transition. Although the pathological context differs, the metabolic findings of aberrant glycolysis and impaired fatty acid oxidation are consistent with DKD.

Empagliflozin is a clinically impactful agent, with the EMPA-REG trial demonstrating significant reductions in renal complications among high-risk diabetic patients [107,108]. Beyond glycemic control, its mechanistic insights reveal a direct link to glycolysis regulation. In STZ-induced diabetic CD-1 mice, empagliflozin reverses kidney fibrosis and EMT. Empagliflozin restores suppressed SIRT3 expression and corrects aberrant glycolysis, which is characterized by HIF-1α accumulation, HK2 induction, and PKM2 dimer formation. In HK-2 cells cultured in high-glucose media, empagliflozin or SGLT2 knockdown suppresses EMT, restores SIRT3, and normalizes glycolytic activity [109].

Collectively, these findings highlight that SGLT2 inhibitors not only improve renal outcomes clinically but also directly target the regulation of glycolytic pathways.

### 5.2. PKM2 Agonists

Recent studies indicate that pharmacological activation of PKM2 tetramers with agents such as TEPP-46 and DASA-58 counteracts hyperglycemia-induced toxic metabolite accumulation in renal cells, restores metabolic homeostasis, and confers renoprotective effects. TEPP-46 is a small-molecule activator that promotes PKM2 tetramer formation by stabilizing the interactions between its subunits, thereby enhancing PK activity [110]. TEPP-46 modulates glycolytic activity in DKD by inhibiting hypoxia response element transcriptional activity and suppressing the aberrant expression of glycolysis markers HK1 and HK2. Importantly, TEPP-46 treatment does not alter blood glucose levels, body weight, or kidney/body weight ratio in a STZ-induced DKD mouse model, indicating that its effects are independent of systemic glycemic control. Consistent with changes in hexokinase expression, TEPP-46 reduces lactate accumulation [72]. TEPP-46 also counteracts oxidative stress-induced cellular senescence. In primary renal tubular cells, hydrogen peroxide triggers senescence markers (p16, p21, p53) and lactate overproduction, which can be suppressed by TEPP-46 co-incubation. In db/db mice, TEPP-46 administration alleviates apoptosis and fibrosis, with a tendency to lower senescence-associated molecules in the kidney [111]. These findings suggest that TEPP-46 exerts renoprotective effects by directly targeting glycolysis to correct metabolic imbalance independent of blood glucose levels, while simultaneously suppressing oxidative stress-induced senescence and inflammation.

The novel imidazopyrimidine-based derivative 15n is a recently developed potent PKM2 activator with an AC50 of 90 nM [112]. Treatment with 15n enhances PKM2 tetramer formation, thereby reducing EMT and fibrosis progression. 15n attenuates DKD progression, accompanied by decreased serum urea and creatinine levels compared to untreated diabetic mice [113].

### 5.3. Chinese Medicine

Many traditional Chinese medicine (TCM) formulas ameliorate DKD through glycolytic mechanisms. Fufang Zhenzhu Tiaozhi (FTZ) demonstrates broad metabolic and renal benefits in DKD, including reductions in blood glucose, lipids, proteinuria, and serum creatinine. FTZ also improves glucose tolerance and renal pathology. Mechanistically, FTZ exerts a distinct influence on glycolysis. Pinusolidic acid, one of its active components, shows strong binding affinity to multiple pockets of SGLT2. Through this binding activity, FTZ treatment significantly downregulates SGLT2 expression as well as glycolysis-related proteins such as glucose transporter 2 (GLUT2), PKM2, and HK2 [114]. LDH activities also decrease to reduce the amount of lactate [114]. Shenhua tablet (SHT) demonstrates therapeutic benefits in DKD by lowering 24 h urinary protein excretion, regulating blood glucose, improving renal tissue structure, and protecting renal function. Mechanistically, SHT exerts its effects by modulating glycolysis in macrophages, reducing polarization toward the proinflammatory M1 phenotype, and suppressing activation of the HIF-1α/PKM2 signaling pathway [115]. The modified Shen–Yan–Fang–Shuai formula (M-SYFSF) significantly improves renal function in DKD, reduces serum creatinine and proteinuria while ameliorating histopathological changes. Mechanistically, modified Shen-Yan-Fang-Shuai formula suppresses HIF-1α expression and nuclear translocation, leading to downregulation of glycolytic enzymes (HK2, LDH, PDK1) and a metabolic shift from glycolysis toward oxidative phosphorylation [116].

Apart from using TCM formulations, active monomers derived from TCM also play a critical role in modulating glycolysis and protecting renal function in DKD. Shikonin protects podocytes by reducing PKM2 nuclear translocation, thereby preventing oxidative stress and NF-κB activation. Through this mechanism, shikonin restores glycolysis and maintains podocyte integrity [86]. Stachyose binds SGLT2 at the Tyr270 site with high affinity, promoting its degradation and reversing metabolic reprogramming. In HK-2 cells, stachyose restores fatty acid oxidation, suppresses glycolysis, reduces lipid deposition, and improves mitochondrial function. In db/db mice, stachyose enhances renal function and alleviates fibrosis [117]. Gallic acid is also an important active monomer commonly found in TCM. Methylglyoxal is a glycolytic by-product and contributes to advanced glycation, oxidative stress, and fibrosis [118]. In a methylglyoxal-induced mouse model, gallic acid reduces albuminuria, restores glyoxalase 1 and antioxidant enzymes, shifts the fibrosis-associated microRNAs and miR-192/miR-204 antifibrotic miR-29a [119], and counteracts the effects of methylglyoxal. Thus, gallic acid primarily limits glycolytic by-product toxicity instead of directly inhibiting glycolytic flux.

Collectively, TCM formulas and extracted active monomers play important renoprotective roles and show great potential for DKD management and biomarker discovery.

### 5.4. Other Compounds and Interventions

Several interventions converge on glycolysis by restoring renal substrate metabolism and systemic metabolic reprogramming. The concentration of the endogenous tetrapeptide N-acetyl-seryl-aspartyl-lysyl-proline (AcSDKP) increases with angiotensin-converting enzyme inhibition. AcSDKP restores SIRT3 and mitochondrial fatty acid oxidation, suppresses abnormal renal glucose metabolism, and attenuates metabolism-linked mesenchymal transition and fibrosis. Conversely, inhibition of AcSDKP synthesis aggravates metabolic disruption and fibrosis-related renal injury [120]. A complementary approach is to reactivate oxidative metabolism in tubular cells. The selective PPARα/δ dual agonist H11 normalizes glycolytic alterations while promoting fatty acid oxidation and branched-chain amino acid degradation in db/db mice and high-glucose–exposed tubular epithelial cells, thereby ameliorating renal injury [121]. As discussed above, Enarodustat mitigates the accumulation of glycolytic and tricarboxylic acid-cycle metabolites and reduces oxidative stress in early DKD [38]. Together, these studies suggest that therapeutic potential can arise from rebalancing glycolysis by restoring mitochondrial substrate oxidation.

Other compounds act on the metabolites of glycolysis. The tricyclic matrine derivative 17a increases glucose consumption and glycolysis while suppressing aerobic glucose oxidation in HepG2 cells and L6 skeletal muscle cells, thereby reducing mitochondrial electron transport chain burden and exerting a renoprotective effect. Separately, in KK-Ay mice, 17a lowers blood glucose, glycated hemoglobin, AGEs, and urinary protein and improves renal function and pathology, indicating renoprotective effects [122]. This pattern indicates that the way glycolysis is regulated in DKD is dependent on the context. In the case of the tricyclic matrine derivative, even though glycolytic throughput is increased, redirecting glucose disposal to improve mitochondrial function can be compensatory and beneficial to DKD management.

Lifestyle adjustment intervention also provides strong human translational evidence. Weight loss is accompanied by lower albuminuria and glomerular hyperfiltration and improvement in early kidney injury. Kidney transcriptomics show coordinated suppression of glycolysis/gluconeogenesis and tricarboxylic acid-cycle gene programs together with reduced mTORC1 and JAK/STAT signaling, alongside the upregulation of protective pathways like AMPK/FOXO3 through the altered nutrient input after the surgery (e.g., gastrectomy) [123]. These observations support systemic metabolic reprogramming as a way to renal protection. However, this notion requires further validation because of the limited biopsy cohort (*n* = 5) and absence of a nonsurgical control group. Therefore, these findings should be regarded as indirect hypothesis-generating evidence of treatment-responsive renal metabolic remodeling. They do not establish suppression of glycolysis as the mechanism of renal benefit or support bariatric surgery as a glycolysis-targeted DKD therapy. Apart from lifestyle adjustment and surgical interventions, certain peptides also show therapeutic potential. The synthetic CFB-derived peptide also exhibits great potential as an antifibrotic intervention in DKD management through HIF-1α/glycolysis axis suppression [124].

Overall, these approaches broaden glycolysis-directed therapy from glycolysis inhibition, such as single-enzyme inhibition, to restoration of metabolism, systemic metabolic reprogramming through surgery, and biomarker discovery.

**Table 1:** Therapeutic strategies targeting glycolysis, glycolysis-related pathways, and metabolites in DKD.

Category	Agent/Intervention	Molecular Targets and Their Downstream Proteins	Evidence	Direct/Indirect to Glycolysis
SGLT2 inhibitor	Canagliflozin [104]	Glycine	Clinical/translational evidence	Indirect
	Dapagliflozin [39,106]	SIRT3, PGC-1α, HIF-1α, SGLT2	Preclinical renal evidence	Indirect
	Empagliflozin [109]	SIRT3, HIF-1α, HK2, PKM2, SGLT2	Preclinical mechanistic evidence	Indirect
PKM2 agonist	TEPP-46 [72,111]	PKM2, HIF-1α, HK1, HK2	Preclinical mechanistic evidence	Direct
	15n [113]	PKM2, HIF-1α	Preclinical mechanistic evidence	Direct
Chinese medicine	Fufang Zhenzhu Tiaozhi capsule [114]	SGLT2, GLUT2, HK2, PKM2, LDH	Preclinical in vivo/in silico evidence	Indirect
	Shenhua Tablet [115]	HIF-1α, PKM2, TNF-α, IL-1β, IL-6, MCP-1	Preclinical in vivo evidence	Indirect
	Modified Shen-Yan-Fang-Shuai formula [116]	HIF-1α, HK2, LDH, PDK1	Preclinical mechanistic evidence	Indirect
	Shikonin [86]	SIRPα, PKM2, NF-κB	Preclinical mechanistic evidence	Indirect
	Stachyose [117]	SGLT2, AMPK	Preclinical target-validation evidence	Indirect
	Gallic acid [119]	Nrf2, miR-192, miR-204, miR-29a	Preclinical in vivo downstream-toxicity model	Indirect
Peptide	N-acetyl-seryl-aspartyl-lysyl-proline (AcSDKP) [120]	SIRT3	Preclinical in vivo evidence	Indirect
	CFB-derived peptides [124]	HIF-1α, HK2, LDHA	Human biomarker discovery plus in vitro evidence	Indirect
Hypoxia-inducible factor prolyl hydroxylase inhibitor	Enarodustat [38]	HIF-1α	Preclinical in vivo/omics evidence	Indirect
PPARα/δ dual agonist	H11 [121]	PPARα, PPARδ	Preclinical mechanistic evidence	Indirect
Tricyclic matrine compound	17a [122]	----	Nonrenal in vitro plus preclinical in vivo evidence	Indirect

Abbreviations: AMPK, AMP-activated protein kinase; mTOR, mechanistic target of rapamycin; SIRT3, sirtuin 3; PGC-1α, peroxisome proliferator-activated receptor gamma coactivator 1-alpha; HIF-1α, hypoxia-inducible factor 1-alpha; HK2, hexokinase 2; PKM2, pyruvate kinase M2; HK1, hexokinase 1; SGLT2, sodium-glucose cotransporter 2; GLUT2, glucose transporter 2; LDH, lactate dehydrogenase; TNF-α, tumor necrosis factor-alpha; IL-1β, interleukin-1 beta; IL-6, interleukin-6; MCP-1, monocyte chemoattractant protein-1; LDHA, lactate dehydrogenase A; PDK1, pyruvate dehydrogenase kinase 1; SIRPα, signal regulatory protein alpha; NF-κB, nuclear factor kappa-light-chain-enhancer of activated B cells; Nrf2, nuclear factor erythroid 2–related factor 2; miR-192, microRNA-192; miR-204, microRNA-204; miR-29a, microRNA-29a; PPARα, peroxisome proliferator-activated receptor alpha; PPARδ, peroxisome proliferator-activated receptor delta.

## 6. Glycolytic Reprogramming as a Source of Potential Diagnostic Biomarkers in DKD

A number of potential diagnostic biomarkers emerge from glycolytic reprogramming, as summarized in Table 2.

### 6.1. Humoral Biomarkers

Lactate has received strong clinical support as one of the glycolysis-related biomarkers in DKD. Multiple clinical studies demonstrate that patients with DKD have higher urinary lactate than age- and sex-matched controls. In addition, patients with the third tertile of urinary lactate-to-creatinine ratio experience a more rapid decline in eGFR than those in the first tertile [125]. Euglycemic and hyperglycemic clamp studies in type 1 diabetes further show that both plasma and urinary lactate rise with glucose, while human DKD biopsy data localize upregulated glycolytic genes to proximal tubular cells [125]. These convergent observations link a readily measurable urinary metabolite to renal glycolytic activation, tubular glucose handling, and subsequent loss of kidney function.

Independent clinical-pathological evidence supports the same lactate-LDHA axis. In patients with DKD, urinary lactate and renal LDHA expression are negatively correlated with eGFR [126]. A cross-sectional study including 65 healthy controls, 48 patients with diabetes but no kidney disease, and 60 patients with DKD also identifies a perturbation of glycolysis and the tricarboxylic acid cycle, with increased DHAP and decreased succinyl-CoA synthetase as potential biomarkers of tubular mitochondrial injury [127]. Together, these findings establish urinary lactate as a clinically relevant biomarker of glycolytic activation in DKD, while complementary assessment of upstream intermediates and mitochondrial readouts helps separate excessive glycolysis from impaired pyruvate oxidation.

Nevertheless, the specificity of these metabolites requires careful evaluation. It has been shown in 40 young type 1 diabetic persons presented with diabetic ketoacidosis (DKA), urinary glucose and lactate are highest within 0–8 h after treatment begins and decrease during recovery. These findings indicate that metabolites such as glucose and lactate are non-specific and reflect systemic metabolic stress rather than kidney specific injury. Their elevation in DKA demonstrates that lactate does not signify DKD on its own. Therefore, kidney specific metabolic responses need to be defined before these metabolites can be considered as true biomarkers for DKD. Likewise, serum glycerol-3-phosphate (G3P), a glycolysis-derived metabolite, is found to be increase along with C-terminal FGF23 in the studied cohort. Although G3P closely engage in glycolysis and thereby bears a potential link to DKD progression, this finding likely reflects kidney failure and disturbed mineral metabolism rather than DKD in particular because of the cohort (non-diabetic patients receiving hemodialysis) in this study [128]. While humoral biomarkers may be useful in DKD diagnosis and thus in determining DKD management measures, biomarkers such as urinary lactate, DHAP, and G3P must be evaluated and interpreted cautiously with concordant blood glucose levels, DKA history, concurrent drugs, and renal function before final conclusion and decision-making.

Urinary peptidomics provides another approach to identify key peptides as biomarkers of DKD. In a discovery cohort including 3 healthy controls, 6 patients with diabetes without proteinuria, and 15 patients with DKD, capillary electrophoresis-tandem mass spectrometry has identified 4331 urinary peptides. Eight peptides were observed to vary with disease severity. Among them, two complement factor B (CFB)-derived peptides are increased in DKD, possibly warranting further evaluation as candidate biomarkers of DKD severity. A synthetic peptide derived from complement factor B alleviated renal fibrosis by suppressing the HIF-1α/glycolysis axis, making it a potential humoral biomarker of DKD progression and a therapeutic target for DKD management [124]. However, this discovery study was limited by its small sample size (*n* = 24) and the screening of 4331 peptides without a reported correction for multiple testing [124]. Since no independent validation cohort was included and clinical biomarker qualification generally requires cross-cohort reproducibility, standardized reporting, and longitudinal validation, the eight severity-associated peptides should be considered exploratory candidates rather than validated diagnostic or prognostic biomarkers [124].

Metabolic profiles offer comprehensive measurement of metabolites such as amino acids and sugars which may also serve as candidates for pharmacodynamic monitoring in DKD. As reported in the same clinical cohort described previously, 12 weeks of canagliflozin treatment reduced albuminuria and shifted the plasma metabolome from glycolysis toward fatty acid oxidation [104]. This reversibility is biologically informative because it links clinical improvement with correction of metabolic reprogramming and potential DKD improvement. However, treatment-responsive changes in metabolic pathways do not necessarily benefit the kidneys, and their value for predicting long-term renal outcomes remains to be established.

### 6.2. Proteomic, Transcriptomic, and Extracellular-Vesicle Biomarkers

The evidence summarized here concerns changes in protein or transcript abundance rather than alterations in DNA sequence or genomic structure. In primary human proximal tubular cells, androgen exposure alters the abundance of energy-metabolism proteins, including glucose-6-phosphate isomerase (GPI). Consistently, male diabetic mice show higher renal abundance of GPI and related metabolic proteins than females [80]. These proteomic findings identify GPI as a candidate protein biomarker, not a genomic biomarker, and highlight the need to consider sex when evaluating protein-based biomarkers and defining reference thresholds. In a paired-biopsy study of five young people with type 2 diabetes and severe obesity, kidney transcriptomic analysis before and 12 months after vertical sleeve gastrectomy showed reduced expression of gene sets related to glycolysis/gluconeogenesis and the tricarboxylic acid cycle in proximal tubular and thick ascending limb cells [123].

EVs, of which exosomes are one subtype, may provide a means of sampling cell-specific metabolic communication without kidney biopsy. In preclinical experiments, EVs released by human serum albumin (HSA)-stimulated tubular epithelial cells were taken up by macrophages and stabilized HIF-1α, thereby increasing macrophage glycolytic activity, inflammatory signaling, and profibrotic responses [94]. This evidence supports a mechanism of EV-mediated tubular–macrophage crosstalk but does not identify a genomic alteration or validate a circulating or urinary EV biomarker in patients. Clinical translation will require identification of the relevant EV cargo, standardized vesicle isolation, normalization, and quantification, and validation in independent patient cohorts.

**Table 2:** Glycolysis-related biomarker candidates and their evidence status in DKD.

Biomarker	Matrix	Cohort	Comparator	Effect	Validation Status
**Urinary lactate and lactate/creatinine ratio** [125]	Urine	HUNT3 participants with T2D; SMART2D and CRIC cohorts	DKD vs. age- and sex-matched controls; highest vs. lowest lactate/creatinine tertile	Urinary lactate was higher in DKD; the highest tertile was associated with faster eGFR decline.	Multicohort human association with prognostic replication; clinical cutoff, specificity, and assay validation remain outstanding.
**Glucose-responsive lactate** [125]	Plasma and urine; kidney biopsy	Participants with T1D undergoing glucose clamps; human DKD biopsy datasets	Hyperglycemic vs. euglycemic clamp conditions	Plasma and urinary lactate increased with glucose; glycolytic transcripts localized mainly to proximal tubular cells.	Supportive physiological and tissue evidence, but not an independent diagnostic or prognostic validation study.
**Urinary lactate and renal LDHA abundance** [126]	Urine and kidney tissue	Patients with DKD	Associations across kidney-function and tissue-injury measures	Urinary lactate and renal LDHA abundance were negatively correlated with eGFR.	Single clinical-pathological study with mechanistic model support; no independent patient-cohort validation reported.
**DHAP and succinyl-CoA synthetase activity** [127]	Serum	65 healthy controls, 48 patients with diabetes without kidney disease, and 60 patients with DKD	DKD vs. diabetes controls and healthy controls	DHAP was increased and succinyl-CoA synthetase activity was decreased in DKD, consistent with glycolytic and mitochondrial disturbance.	Single cross-sectional cohort with internal targeted confirmation after metabolomic discovery; no external validation cohort.
**Urinary glucose and lactate during DKA** [129]	Urine	40 young people with T1D presenting with DKA	Early treatment (0–8 h) vs. recovery	Both metabolites were highest early after treatment began and declined during recovery.	Not a DKD validation cohort; demonstrates confounding by acute glycemia and acid-base status.
**Glycerol-3-phosphate** [128]	Serum	Nondiabetic patients receiving hemodialysis	Association with C-terminal FGF23	Serum glycerol-3-phosphate increased with C-terminal FGF23.	Human association but not DKD-specific; likely reflects kidney failure and mineral-metabolism disturbance.
**CFB-derived urinary peptides** [124]	Urine	Discovery cohort: 3 healthy controls, 6 patients with diabetes without proteinuria, and 15 patients with DKD (*n* = 24)	Clinical groups and DKD-severity categories	Eight of 4331 peptides varied with severity; two CFB-derived peptides were increased in DKD. A synthetic CFB peptide suppressed HIF-1α/glycolysis signaling in tubular cells.	Exploratory discovery only: no reported multiplicity correction, incomplete effect reporting, and no independent validation cohort.
**Canagliflozin-responsive metabolic signature** [104]	Plasma metabolome	35 patients with T2D and DKD treated for 12 weeks	After treatment vs. baseline	Albuminuria decreased and the plasma metabolome shifted from glycolysis toward fatty-acid oxidation.	Single-arm pharmacodynamic evidence; not validated for diagnosis, prognosis, or long-term renal-outcome prediction.
**GPI protein abundance** [78]	Primary human proximal-tubular-cell proteome and diabetic mouse kidney tissue	Androgen-exposed human tubular cells and male vs. female diabetic mice	Androgen exposure vs. control; male vs. female diabetic mice	Androgen exposure altered GPI abundance; renal GPI and related metabolic proteins were higher in male diabetic mice.	Preclinical proteomic candidate; no patient biofluid assay or independent clinical validation.
**Tubular epithelial cell-derived EV glycolytic signal** [94]	Cell-culture EV preparation and recipient macrophages	HSA-stimulated tubular epithelial cells with macrophage uptake experiments	EV-exposed vs. control macrophages	EV uptake stabilized HIF-1α and increased macrophage glycolysis, inflammatory signaling, and profibrotic responses.	Preclinical mechanistic evidence only; relevant EV cargo and a circulating or urinary patient assay have not been defined or validated.

Abbreviations: CFB, complement factor B; Cr, creatinine; DHAP, dihydroxyacetone phosphate; DKA, diabetic ketoacidosis; EV, extracellular vesicle; GPI, glucose-6-phosphate isomerase; HSA, human serum albumin; LDHA, lactate dehydrogenase A; SCS, succinyl-CoA synthetase; TCA, tricarboxylic acid; T1D, type 1 diabetes; T2D, type 2 diabetes.

## 7. Conclusions and Future Perspectives

Glycolysis serves as both a mechanistic driver and a potential biomarker source in DKD. Current evidence indicates that glycolysis-derived signatures may provide clinically relevant information, yet none are sufficiently validated to replace established measures such as urinary albumin-to-creatinine ratio or eGFR [4]. Urinary lactate shows a direct association with subsequent eGFR decline, while the use of other humoral, genomic, and exosome-based biomarkers remains at an early stage that requires further validation. Prospective studies are needed to clarify whether glycolysis-related biomarkers improve risk stratification and clinical decision-making beyond conventional parameters. To achieve this, standardized protocols for sample collection, normalization, and patient stratification are important, together with serial sampling and hard renal outcomes to distinguish diagnostic, prognostic, and pharmacodynamic roles. These steps help determine whether glycolytic signatures represent true biomarkers of DKD or context-dependent correlates of hyperglycemia and reduced kidney function.

Therapeutic modulation of glycolysis in DKD is equally complex. A substantial proportion of therapeutic interventions aim to suppress glycolytic flux in order to limit lactate accumulation and downstream toxicity. In addition, certain compounds target the downstream consequences of glycolytic activity, particularly the by-products that drive oxidative stress and fibrosis. Gallic acid illustrates this approach by counteracting methylglyoxal toxicity, restoring glyoxalase 1 activity, enhancing antioxidant defenses, and shifting fibrosis-associated microRNAs toward antifibrotic profiles [119]. In contrast, the tricyclic matrine derivative 17a represents a distinct paradigm. 17a enhances glucose consumption and glycolysis while limiting aerobic glucose oxidation, thereby reducing mitochondrial electron transport chain burden [122]. This metabolic rerouting improves renal function and pathology in diabetic models, accompanied by reduction in blood glucose, glycated hemoglobin, advanced glycation end products, and proteinuria. These different examples highlight the fact that therapeutic strategies may either involve inhibition or enhancement of glycolysis, depending on whether the goal is to limit toxic by-products or to relieve mitochondrial stress. A combination approach may also be useful, but require further investigation of the therapeutic thresholds in a cell type-specific and context-dependent manner.

PKM2 illustrates a paradoxical role in DKD, with opposing outcomes depending on its structural state. In its dimeric form, PKM2 exhibits reduced catalytic activity, leading to insufficient pyruvate production and excessive lactate accumulation. This imbalance fuels oxidative stress, inflammation, and fibrosis, thereby aggravating renal injury. In contrast, stabilization of PKM2 in its tetrameric form restores enzymatic activity and metabolic homeostasis. This shift corrects abnormal glycolytic flux, reduces lactate overflow, and alleviates mitochondrial dysfunction. In addition, tetrameric PKM2 activation is associated with suppression of oxidative stress-driven senescence and inflammation, further contributing to renoprotection [72]. As such, PKM2 is not simply a glycolytic enzyme but a metabolic switch whose conformational dynamics determine whether it drives disease progression or confers protection. Therefore, future therapeutic strategies need to focus less on broadly inhibiting or enhancing glycolysis, but more on selectively modulating PKM2’s structural state to achieve context-specific benefits in DKD.

Another dimension is the cell-type-specific regulation of glycolysis. Differentiated cell types exhibit distinct glycolytic responses to hyperglycemia, hypoxia, and inflammatory stress. For instance, podocytes exhibit PKM2/NF-κB activation under diabetic stress, which impairs aerobic glycolysis and contributes to metabolic vulnerability, while tubular epithelial cells under albumin stress release extracellular vesicles that reprogram macrophage glycolysis and fibrosis. These findings emphasize that glycolysis in DKD is not a uniform process but a network of cell-specific metabolic adaptations. Therapeutic interventions may therefore need to be tailored to specific cellular compartment most relevant to disease progression. Taken together, the specific novelty of this review lies in integrating a cell-type-resolved organization of glycolytic alterations across renal and immune compartments with a context-dependent therapeutic framework.

Despite these advances, several challenges remain before glycolysis can be fully translated into clinical diagnostic or therapeutic tools. The development of biomarkers requires rigorous external validation, standardized sampling protocols, and integration with existing risk models. Therapeutic modulation faces the difficulty of balancing inhibition versus enhancement, avoiding systemic metabolic disruption, and accounting for heterogeneity across patient populations. The interplay between glycolysis and mitochondrial dysfunction, PTMs, and immune-inflammatory signaling further complicates the identification of causal versus correlative pathways. Addressing these challenges likely depends on multi-omics approaches, longitudinal patient cohorts, and translational studies that bridge mechanistic insights with clinical endpoints.

## Data Availability

No new data were created or analyzed in this study. Data sharing is not applicable to this article.

## Abbreviations

The following abbreviations are used in this manuscript:

α-SMA	Alpha-smooth muscle actin
AcSDKP	N-acetyl-seryl-aspartyl-lysyl-proline
AGEs	Advanced glycation end-products
AKT	Protein kinase B
ALDOB	Aldolase type B
AMPK	AMP-activated protein kinase
ARAP1	ArfGAP with RhoGAP domain, ankyrin repeat and PH domain 1
ARAP1-AS2	ARAP1 antisense RNA 2
ATPIF1	ATP synthase inhibitory factor subunit 1
CD36	Cluster of differentiation 36
CKD	Chronic kidney disease
COUP-TFII	Chicken ovalbumin upstream promoter transcription factor II
DENV-2	Dengue virus serotype 2
DHAP	Dihydroxyacetone phosphate
DKD	Diabetic kidney disease
DHT	Dihydrotestosterone
eGFR	Estimated glomerular filtration rate
EMT	Epithelial–mesenchymal transition
EndoMT	Endothelial-to-mesenchymal transition
ERA	Endothelin receptor antagonist
ESRD	End-stage renal disease
EVs	Extracellular vesicles
FBP	Fructose-1,6-bisphosphate
FSP-1	Fibroblast-specific protein-1
GEC	Glomerular endothelial cell
GPI	Glucose-6-phosphate isomerase
GNPNAT1	Glucosamine-phosphate N-acetyltransferase 1
GPR87	G protein-coupled receptor 87
H3K14la	Histone H3 lysine 14 lactylation
H3K18la	Histone H3 lysine 18 lactylation
H4K12la	Histone H4 lysine 12 lactylation
HADHA	Hydroxyacyl-Coa dehydrogenase
HIF-1α	Hypoxia-inducible factor 1-alpha
HK1	Hexokinase 1
HK2	Hexokinase 2
HMCs	Human primary mesangial cells
IGFBP5	Insulin-like growth factor binding protein 5
IL-1β	Interleukin-1 beta
IL-22	Interleukin-22
IREB2	Iron-responsive element-binding protein 2
KLF5	Krüppel-like factor 5
LDH	Lactate dehydrogenase
LDHA	Lactate dehydrogenase A
lncRNA	Long non-coding RNA
MRA	Mineralocorticoid receptor antagonists
mtROS	Mitochondrial reactive oxygen species
NAD	Nicotinamide adenine dinucleotide
NLRP3	NOD-, LRR- and pyrin domain-containing protein 3
OXPHOS	Oxidative phosphorylation
PDK1	Pyruvate dehydrogenase kinase 1
PDK4	Pyruvate dehydrogenase kinase 4
PFK1	Phosphofructokinase 1
PFKP	Phosphofructokinase platelet type
PFKFB2	6-phosphofructo-2-kinase/fructose-2,6-bisphosphatase 2
PFKFB3	6-phosphofructo-2-kinase/fructose-2,6-bisphosphatase 3
PGC-1α	Peroxisome proliferator-activated receptor gamma coactivator 1-alpha
PK	Pyruvate kinase
PKM1	Pyruvate kinase M1
PKM2	Pyruvate kinase M2
PTM	Post-translational modification
RAAS	Renin–angiotensin–aldosterone system
RAGE	Receptor for advanced glycation end-products
ROS	Reactive oxygen species
RUNX1	Runt-related transcription factor 1
SAE1	SUMO-activating enzyme subunit 1
SGLT2	Sodium-glucose cotransporter 2
SIRPα	Signal regulatory protein alpha
S1PR1	Sphingosine 1-phosphate receptor 1
SIRT3	Sirtuin 3
SIRT5	Sirtuin 5
SIRT6	Sirtuin 6
SLC2A1	Solute carrier family 2 member 1
Smad4	Mothers against decapentaplegic homolog 4
STZ	Streptozotocin
SUMOylation	Small ubiquitin-like modifier conjugation
TCM	Traditional Chinese medicine
TECs	Renal tubular epithelial cells
TERT	Telomerase reverse transcriptase
TGF-β	Transforming growth factor beta
TRIM65	Tripartite motif-containing protein 65
T1DM	Type 1 diabetes mellitus
T2DM	Type 2 diabetes mellitus
UBR5	Ubiquitin protein ligase E3 component N-recognin 5
VEGF	Vascular endothelial growth factor
WTAP	Wilms tumor 1-associating protein
YY1	Yin and Yang 1 transcription factor
